# Cytoplasmic TERT Associates to RNA Granules in Fully Mature Neurons: Role in the Translational Control of the Cell Cycle Inhibitor p15INK4B

**DOI:** 10.1371/journal.pone.0066602

**Published:** 2013-06-18

**Authors:** Francesca Iannilli, Francesca Zalfa, Annette Gartner, Claudia Bagni, Carlos G. Dotti

**Affiliations:** 1 Center for Human Genetics, Katholieke Universiteit Leuven, Leuven, Belgium; 2 VIB Center for the Biology of Disease – VIB, Leuven, Belgium; 3 University of Rome, “Campus Bio-medico” CIR Department, Rome, Italy; 4 Centro de Biología Molecular Severo Ochoa, CSIC/UAM, Madrid, Spain; University of Turin, Italy

## Abstract

The main role of Telomerase Reverse Transcriptase (TERT) is to protect telomere length from shortening during cell division. However, recent works have revealed the existence of a pool of TERT associated to mitochondria, where it plays a role in survival. We here show that in fully differentiated neurons the largest pool of cytoplasmic TERT associates to TIA1 positive RNA granules, where it binds the messenger RNA of the cyclin kinase inhibitor p15INK4B. Upon stress, p15INK4B and TERT dissociate and p15INK4B undergoes efficient translation, allowing its pro-survival function. These results unveil another mechanism implicated in the survival of fully differentiated neurons.

## Introduction

Senescence, or biological aging, can be defined as the accumulation of changes in the biology of an individual over time, correlated with increased susceptibility to disease and mortality. Much of the changes that occur with aging are the consequence of a process known as cellular senescence: i.e. irreversible loss of replicative capacity after certain number of divisions[1]. We now know that this time-associated replicative impairment is due, to a large extent, to the shortening of telomeres which occur with each division[2]. In turn, telomere shortening is due to a combination of causes, including oxidative damage in guanine triplets[3], which are abundant at telomeric ends, and the decrease in the activity of the telomere shortening-inhibiting enzyme telomerase reverse transcriptase (TERT)[4]. In proliferative cells, with the exception of germinal cells, stem cells and committed progenitors, TERT activity decreases after 50–60 division cycles bringing cells into the replicative senescence state, i.e. appearance of permanently post-mitotic cells which gradually start to lose function: the aging process.

Neurons are a most suitable cellular system to dissect the mechanisms implicated in survival-function homeostasis in the post-mitotic stage. In fact, neurons become permanently arrested in the G0 phase early in development and from this time (and especially after the establishment of synaptic activity), these post-mitotic cells are exposed to the constant presence of stress by-products derived from the intense metabolic needs of the brain. Still, the total number of neurons does not significantly decrease with age, implying that a major effort in the biology of these cells is dedicated to warrant cell survival [5] [6] [7]. Consistent with their post-mitotic quiescence, telomere length in neurons does not change with age[8]. However, TERT does remains abundant in the fully differentiated neuron[9], suggesting that neuronal TERT may play a telomere-independent role. In agreement with this possibility, work in cancer cells[10] and in experimental paradigms of brain excitotoxicity[9] [11] have suggested a mitochondria-associated, pro-survival function.

In this work, we have investigated the possibility that a similar mechanism may be part of the constitutive survival machinery of aging neurons. Our data show that TERT plays a pro-survival role in fully differentiated neurons through its association to RNA granules, where it contributes to the translational control of the pro-survival gene p15INK4B (Cyclin-dependent kinase inhibitor 2B).

## Materials and Methods

### Primary culture of hippocampal neurons

Primary cultures were prepared from Wistar rat fetuses at embryonic day 18–19 as described by Kaech and Banker[12]. The pregnant mother is killed by anaesthetization and cervical dislocation and the embryos are removed from the uterus under sterile conditions. The hippocampi of the embryos are dissected and dissociated by trypsinization.

### Antibodies

For protein detection, the following antibodies were used: rabbit anti-TERT (1∶1000, Santa Cruz, Acris Antibody and LifeSpan BioSciences, Inc.), mouse anti-Tubulin (Cell Signaling), p58, ribophorin 1 (both gift from Wim Annaert, KULeuven), histone 3 (Cell Signaling), TIA1 (Sigma-Aldrich, Santa Cruz Biotechnology), LSM-1 (gift from Tilmann Achsel, KULeuven), P-eIF2α (Cell Signaling), PABP (Santa Cruz Biotechnology). Images were taken with the Fujifilm LAS-3000 system and analyzed with the Image J software (NIH).

### Immunofluorescence Microscopy

Neurons on glass coverslips were incubated with DAPI (Sigma-Aldrich), and rabbit anti-TERT (1:1000, from either Acris Antibody or LifeSpan BioSciences, Inc.). Samples were analyzed on a confocal microscope (Biorad Radiance) and quantification performed using the Mender’s coefficient plugin from NIH ImageJ.

### Immunohistochemistry

Anesthetised mice (black C57BL/6) were perfused intracardially with 10 ml of 0.1 M PBS solution at pH 7.4, followed by 15 ml of ice-cold 4% paraformaldehyde in PBS solution at room temperature. Brains were first submerged in fixative and then in 30% sucrose-PBS solution at 4°C until being frozen in isopentane and cut in 20 µm -thick coronal sections. On the day of the immunohistochemical staining, the sections were placed in a humid chamber, rinsed with PBS and permeabilized with Triton X-100 1% for 30 minutes. After incubation with the blocking solution, the sections were incubated with the primary antibody anti-TERT (LifeSpan BioSciences, Inc.) O.N. (together with the antibody against the subcellular marker) and then with the anti-Rabbit Alexa Fluor 488-conjugated secondary antibody (Invitrogen) and DAPI (Sigma-Aldrich) 1 hr at room temperature.

### Subcellular fractionation

Preparation of different subcellular fractions was made from brains of 23 months old mice (black C57BL/6, n = 4) and rats (Wistar, n = 3) according to Gray and Whittaker’s protocol[13]. All the steps of the fractionation protocol were carried out at 4°C. Briefly, total mouse brains were broken in the homogenization buffer (0.32 M sucrose, 1 mM EDTA, 5 mM HEPES, 1 mM NaV, 1 mM NaF and 25 mM protease inhibitors) using a Teflon/glass homogenizer; 1 ml of the total homogenate was taken apart and the left over centrifuged 3 times, at 1000 g for 10 minutes. The pellet of the third centrifugation corresponds to the nuclear fraction (P1). From the SN of each centrifugation the mitochondrial fraction (P2) was separated using a refrigerated angle-head centrifuge at 17000 g for 55 minutes. The SN was then further centrifuged at 10?5 g for 60 minutes to obtain the microsomal fraction (P3). Finally, the ribosomal fraction (P4) was prepared by centrifuging the SN of P3 at 10?5 g for further 2 hrs. The pellet from each centrifugation was resuspended in 500 µl of STEN-lysis Buffer. The same volume (30 µl) from each fraction was loaded on NuPAGE 4–12% Bis–Tris gels (Invitrogen) after protein denaturation at 70°C for 10 minutes in NuPAGE Sample buffer.

### Polysome gradient

Total mouse brain from 23 months old mice (black C57BL/6, n = 3) was homogenized in 3 ml of lysis buffer (100 mM NaCl, 10 mM MgCl_2_, 10 mM Tris-HCl pH 7.5, 1% Triton-X100, 1 mM dithiothreitol, 40 U/ml RNase inhibitor, 30 µg/ml cicloeximide, 0.5 mM Na-orthovanadate, 5 mM β-glycerophosphate, 10 µg/ml Sigma protease inhibitor). The lysates were incubated 5 min in ice, centrifuged for 5 min at 12,000 g at 4°C, and 500 µl of supernatants (cytoplasmic extract) centrifuged through 15%–50% (w/v) sucrose gradients for 110 min at 37,000 rpm in a Beckman SW41 rotor. Each gradient was collected in 10 fractions. From each fraction the proteins were precipitated with Magic Mix (50% Ethanol, 25% Methanol and 25% Acetone) and analyzed by Western blotting. For Puromycin treatment, before loading on 15%–50% sucrose gradient, brain cytoplasmic extracts were treated with 1 mM Puromycin and 500 mM KCl for 15 min at 4°C.

### Protein and RNA Immunoprecipitation

*IP from brain extracts*: 23 months old mice (black C57BL/6, n = 3) were killed by anaesthetization and cervical dislocation, and the brains were removed under sterile conditions. After homogenization in the same lysis buffer for the polysome analysis (see above), the lysates were incubated 5 min in ice, centrifuged for 5 min at 12,000 g at 4°C. 500 µg of proteins from the SN fraction were loaded on 40 µl protein G sepharose beads (GE Healthcare) for 2 hrs at 4°C to eliminate unspecific bindings. After centrifugation at 1500 rpm for 1 minute, the SN was loaded on new 40 µl protein G sepharose beads conjugated with 5 µg of specific antibodies (hTERT, TIA1 Santa Cruz Biotech) overnight. The day after, the beads were washed 3 times by centrifugation at 1500 rpm for 1 min at 4°C and RNA and proteins were purified and respectively used for PCR amplification or analysed by western blot.

*IP from neurons in culture*: 10 days *in vitro* (DIV) hippocampal neurons were scraped and lysed with Lysis Buffer (50 mM Tris-HCl pH 7.4, 150 mM NaCl, 0.1% Triton, RNase and Protease Inhibitors) using 0.33 mm needle and centrifuged at 2600 rmp for 12 minutes. The SN fraction was treated with benzonase and 200 µg of proteins were loaded on 40 µl protein G sepharose beads following the same protocol described above for the *in vivo* IP.

*RNA purification*: RNAs were first eluited with eluition buffer (0,2 M NaOAc, 1 mM EDTA, 0,2% SDS), heating for 5min at 70°C, then extracted with 1 V of Phenol/Chloroform, and 1 V of Chloroform and finally precipitated with 2,5 V of Absolute EtOH and 20 ug of glycogen (o/n at –20°C). The day after, the samples were centrifuged for 20min at 14000rpm at 4°C, the pellet washed with 70% EtOH and resuspend in 10 ul of H2O. 5 µl of each samples were used for RT-PCR using primers specific for different cell cycle genes. Oligonucleotide primers were synthesized at Invitrogen according to published mRNA sequences (NCBI).

### Cell treatments

To induce oxidative stress, 10 DIV hippocampal neurons in culture, were treated with Arsenite 0.5 mM for 1 hr, at 37°C, 5% CO_2_.

To prevent TERT translocation from the nucleus to the cytosol, 10 DIV TERT overexpressing neurons were treated with 20 ng Leptomycin B (Sigma-Aldrich) for 1hr, at 37°C, 5% CO_2_.

### Scrambled and short hairpin RNA design

Short hairpin (sh) p15INK4B and control construct cloned into lentivirus particles were from Sigma-Aldrich.

The shTERT and scrambled TERT plasmids were generated as reported by Rubinson et al.[14].

The following pairs of oligonucleotides were used:

### shTERT

sense: -5′-P_i_T-GGTGCCTCCTGCAGCGAAA-TTCAAGAGA-TTTCGCTGCAGGAGGCACC-TTTTTTC-3′-

antisense: -3′-A-CCACGGAGGACGTCGCTTT-AAGTTCTCT- AAAGCGACGTCCTCCGTGG-AAAAAAG-AGCTP_i_-5′-

### scrambled TERT

sense: -5′-P_i_T-AAAGCGACGTCCTCCGT GG-TTCAAGAGA-CCACGGAGGACGTCGC TTT-TTTTTTC-3′-

antisense:- 3′- A-TTTCGCTGCAGGAGGCACC- AAGTTCTCT- GGTGCCTCCTGCAGCGAAA-AAAAAAG-AGCTP_i_-5′-

The oligonucleotides for TERT were designed to match the mouse and rat TERT mRNA sequence. Restriction sites XbaI and XhoI were flanking the shTERT and scrambled sequences for the insertion into pLentiLox 3.7 vector (see Cloning section).

### Cloning

The oligonucleotides encoding for scrambled and shTERT were annealed and digested with XbaI and XhoI in order to create the insertion sites for the linear pLentiLox 3.7 vector backbone (Dharmacon, PerBio Science), which contained the U6 promoter upstream of the shTERT and scrambled sequence, an expression cassette composed by the cytomegalovirus promoter (CMV), green fluorescente protein (GFP) and Woodchuck hepatitis virus post-transcriptional regulatory element (WPRE) to monitor transduction efficiency. Ligation was carried out at 16°C overnight and valuated by transformation of DH5α strain of E. Coli competent cells and digestion with the same restriction enzymes used for oligos insertion. The insert was analyzed in 1.5% agarose gel.

### Lentiviral vector generation

293T cells were cultured in Dulbecco’s modified Eagle’s medium (DMEM) supplemented with 2 mM L-Glutamine (Gln), 100 U/ml penicillin, 100 µg/ml streptomycin and 10% heat-inactivated fetal bovine serum (FBS, Invitrogen). Cells were grown in cell factories during viral infection in a special medium 10% Nu-Serum IV (Becton Dickinson). For viral vector production, 90–95% confluent 293T cells were transfected using FuGene 6 reagent (Roche) and viral particles were collected from filtered medium by centrifugation during 2 hrs at 25,000 rpm after transfection. Viral particles were resuspended in 50 µl of 1× PBS, quickly frozen in liquid nitrogen and stored at −80°C.

The second shTERT was from Santa Cruz (product number: L000954).

### TERT over-expression and knock-down

TERT over-expression method: dissociated hippocampal neurons were transfected by neucleofection just before plating using Amaxa Rat Neuron Nucleofector Kit (Lonza) according to the manufacturer's instructions.

TERT knockdown method: 7 DIV hippocampal neurons were incubated for 7 hrs with a lentivial particle expressing the scrambled or sh TERT sequence (dilution 1∶50): the effects of the knock-down were tested 3 days later, at 10 DIV.

### Statistical analysis

Comparisons between groups were performed with the Student T-test and differences were considered significant when p<0.05.

### Ethics Statement

All animal experiments were approved by the Ethics Committee of the K.U.Leuven, Biosafety and Biotechnology.

## Results

Previous work showed that cytoplasmic TERT plays a role in survival in different cell types, including neurons in a cytotoxicity paradigm[9] [11]. Therefore, we performed gain and loss-of-function experiments in cultured hippocampal neurons. Figure S2 shows that TERT knock-down in fully differentiated neurons increased apoptosis. On the contrary, over-expression exerted an anti-apoptotic role (Figure S3). This last effect was prevented by pre-treating cells with the nuclear export inhibitor Leptomycin B (S3b), implying that the pro-survival effect requires TERT that previously accumulated in the nucleus.

Antibody specificity was verified by western blot analysis: Figure S4 (upper panel) shows the concentration-dependent increase of a band at the expected molecular weight. Moreover, knock-down with two different shRNA against TERT and over-expression experiments (Figure S4, lower panel) prove, respectively, the loss and the increase of the target protein.

**Figure 1:**
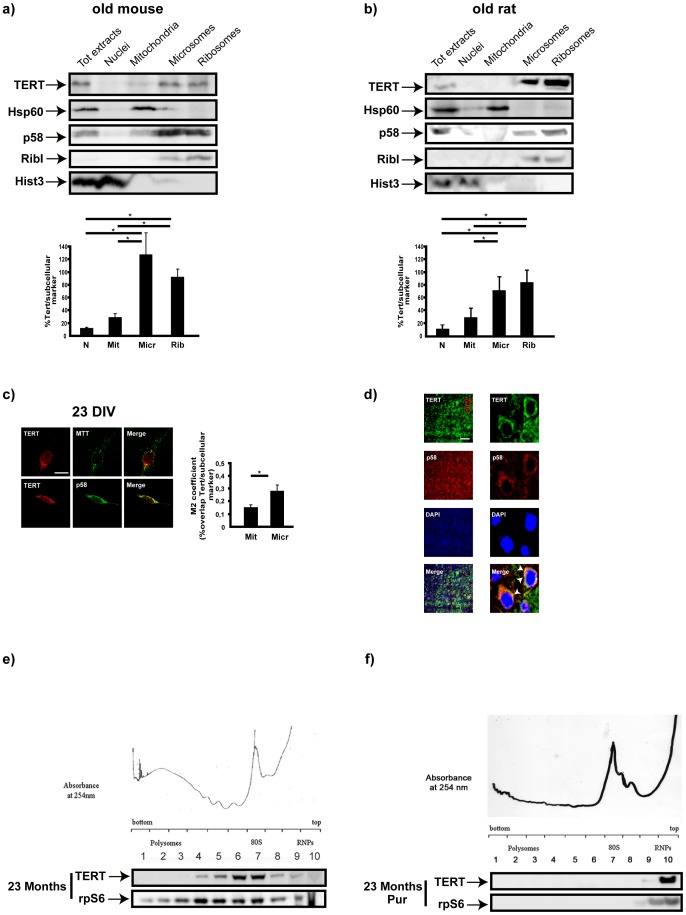
Neuronal TERT is enriched in ribosome-containing compartments *in vivo.* a)-b) Western blot analysis of TERT localization in subcellular fractions from the brains of 23 month-old mice and rats. Hsp60, RibophorinI, p58 and Histone3 are markers of, respectively, mitochondria, ribosomes, microsomes and nuclei. TERT is clearly enriched in the microsome and ribosomal fractions in old mouse brain. Bar graph reflects these differences (mean ± s.d. of four different mice, mean ± s.d. of three different rats, *p<0.05). **c)** Representative confocal images of 23 DIV hippocampal neurons stained for TERT (red) and counterstained with MTT, mitochondrial selective dye (green) and p58 (green), microsomal marker. Bar: 10 µm. Bar graph on the right: quantification of soluble TERT in mitochondria and microsomal fractions; bars represent the mean ± s.d. of three different cultures (*p<0.05). **d)** Representative confocal images of brain slices from 23 months old mice stained with TERT (green), p58 (red) and DAPI (blue). Note the clear cytoplasmic distribution of TERT in the neurons of old mice brain and its concentration in the juxta-nuclear region, coinciding with the enrichment in p58 positive structures (n = 2). Scale bar: 20 µm. **e)** TERT partitioning in polysome gradients from total brain of 23 month-old mice. Note the abundance of TERT in fractions 6–7, corresponding to monosomes and the initiation translational complex (n = 3). **f)** Polysome gradient of old mice brain extracts treated with 1 mM Puromycin. Under this treatment, TERT is displaced to the mRNP fractions. The western blots below each gradient reveal the distribution of TERT and rpS6, used as marker for ribosome-containing fractions (n = 3).

To elucidate the mechanisms behind the pro-survival role of TERT in differentiated neurons, we analyzed TERT cytoplasmic localization using mouse brain sub-cellular fractionation, prepared as in Gray and Whittaker[13]. Western blot analysis from adult mouse and rat brains revealed high levels of the protein in the microsomal and ribosomal fractions (Fig. 1a and 1b). In support of the biochemical data, immunofluorescence microscopy in fully differentiated hippocampal neurons *in vitro* revealed the co-localization of TERT with the p58 protein, a canonical ERGIC (endoplasmic reticulum-Golgi intermediate compartment) marker[15] (Fig. 1c). Similar colocalization was observed *in situ*, in brain sections from old mice (Fig. 1d).

The presence of TERT in RNA-rich organelles and its affinity for G-quadruplex structures, which are also present in RNA[16], motivated us to investigate whether cytoplasmic TERT is in fact associated with RNA/ribosomes. To analyze this possibility, cytoplasmic extracts from mouse brain were fractionated on a 20–50% sucrose gradient (Napoli et al.[17]), and the co-sedimentation of TERT with polysomes/messenger-ribonucleoparticles (mRNPs) was determined by Western blotting[17] [18] [19]. As shown in Fig. 1e, the largest pool of TERT co-sediments mostly with ribosome/monosomes and the 80S initiation complex, partially with RNA bound to two and tree ribosomes and only a small amount of the protein was detected in mRNPs and polysome fractions. To ascertain whether TERT was indeed binding ribosomes, polypepetide elongation was interrupted with puromycin. Fig. 1f shows that this treatment induces a shift of TERT towards the lighter fractions of the gradient (fraction 10), strengthening the notion of a TERT/ribosome association.

**Figure 2:**
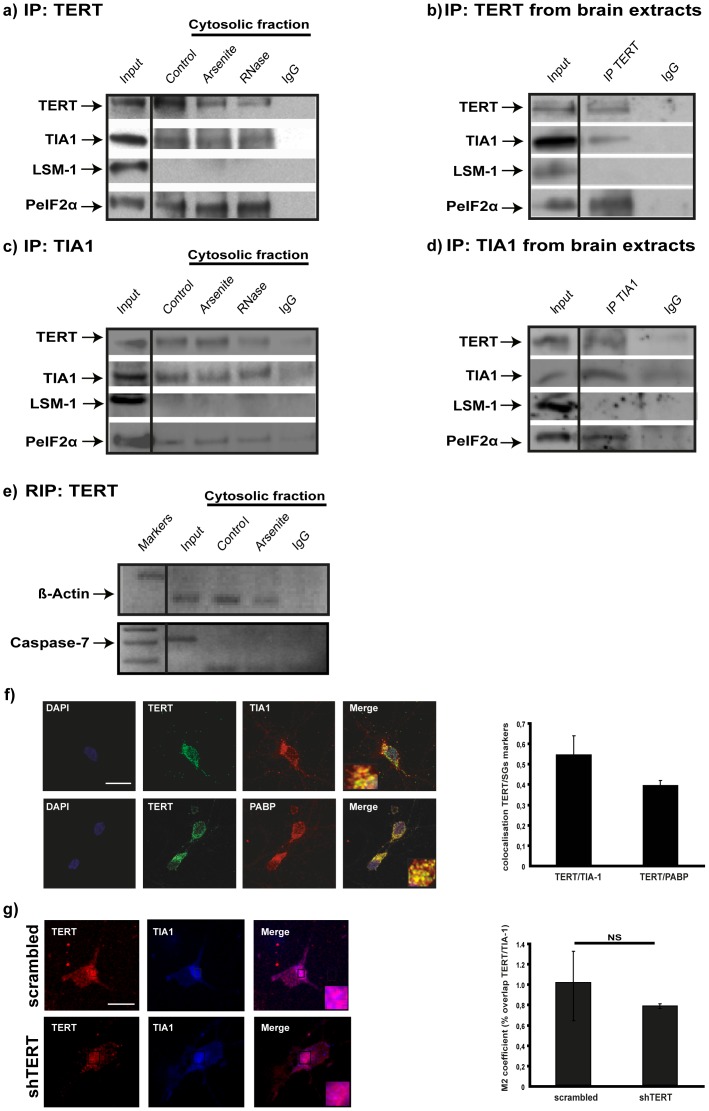
TERT associates to TIA1 –positive granules. **a)** Western blot analysis of TERT immunoprecipitated proteins in extracts from hippocampal neurons maintained *in vitro* for 10 DIV, under basal stress conditions (control) or stressed with arsenite. Note that the two SG markers (TIA1 and P-elF2α) are precipitated whereas LSM-1, a component of PBs is not. RNase treatment does not affect TERT-TIA1 binding (n = 4). **b)** Western blot analysis of TERT immunoprecipitated proteins in extracts from old mice. As in the *in vitro* experiments, TIA1 and P-elF2α are precipitated whereas LSM-1 is not (n = 3). **c)** Western blot analysis of TIA1 immunoprecipitated proteins in extracts from hippocampal neurons maintained *in vitro* for 10 DIV, under basal stress conditions (control) or stressed with arsenite. Again, RNase treatment does not affect TERT-TIA1 binding (n = 4). **d)** Western blot analysis of TIA1 immunoprecipitated proteins in extracts from old mice. Note that TIA1 and P-elF2α are precipitated whereas LSM-1 is not (n = 3). **e)** The known TIA1 target ß-actin mRNA is amplified in RNA purified from TERT immunoprecipitate, whereas another known TIA1 target, Caspase-7, is not. The first lane corresponds to the markers (n = 2). **f)** Confocal microscopy images of neurons double labeled TERT (green)-TIA1 (red) (upper row) and TERT (green)-PABP (red) (lower row). Numerous foci of colocalization exist (quantified in the bar graph: mean ± the s.d. from three different experiments). Scale bar: 10 µm. **g)** Confocal microscopy images of neurons infected with scrambled or shTERT, stained for TERT (red) and counterstained for TIA1 (blue). The reduction in TIA1 labeling is not significant (mean ± the s.d. from three different experiments). Scale bar: 10 µm.

When subjected to environmental stress, cells respond by suspending overall protein synthesis, resulting in the disassembly of polysomes and the stalling of initiation complexes, which become recruited to cytoplasmic *foci* known as stress granules (SGs)[20]. Because cytoplasmic TERT favors neuronal survival (see S2 and S3a) and co-sediments mostly with monosomes and the initiation translational complex, we tested whether TERT is part of SGs. We immunoprecipitated TERT from extracts of hippocampal neurons *in vitro* and determined its possible association with the RNA binding protein TIA1 (T-cell-restricted intracellular antigen-1), a well-known marker of SGs[21]. TIA1 and TERT co-precipitated in the cytosolic fraction from mature hippocampal neurons in culture (Fig. 2a and 2c). A similar result was observed when the immunoprecipitation was performed in brain cytoplasmic extracts from old mice (Fig. 2b and 2d), together suggesting that TIA1 and TERT are in a complex.

Because arsenite increases the number of SGs[22], we treated fully differentiated hippocampal neurons in culture with 0.5 mM arsenite for 60 min. Western blot analysis shows that arsenite treatment does not produce any significant increase in TERT-TIA1 complexes (Fig. 2a and 2c), suggesting that at this stage of neuronal differentiation, the number of complexes is at the upper limit. Moreover, RNase treatment does not affect the binding of TERT to TIA1, demonstrating that the association is RNA-independent. In further support that TERT is part of SGs, co-immunoprecipitation experiments revealed the presence of P-eIF2α, another specific SGs marker[23] (Fig. 2a–2d). On the contrary, LSM-1, a processing body (PB) marker[24], was not found in TERT and TIA1 immunoprecipates (Fig. 2a–2d). As a final confirmation, we performed TERT RNA immunoprecipitation (RIP), followed by RT-PCR with primers for known target genes of TIA1[25]. Fig. 2e shows that the ß-actin mRNA is present in the TERT precipitate, while Caspase-7 mRNA, is not. This result suggests that TERT associates with particular pools of TIA1 granules. The biochemical association of TERT with SG was also confirmed by immunofluorescence microscopy in fully differentiated hippocampal neurons in culture. Fig. 2f shows co-localization TERT-TIA1 and TERT-PABP, another SGs constituent[23]. To test whether TERT is required for SGs formation, we reduced the TERT levels using shRNA, which, however, did not significantly affect the number of TIA1-positive structures (Fig. 2g). These results indicate that TERT associates with different types of TIA1 granules but may not be required for their formation.

**Figure 3:**
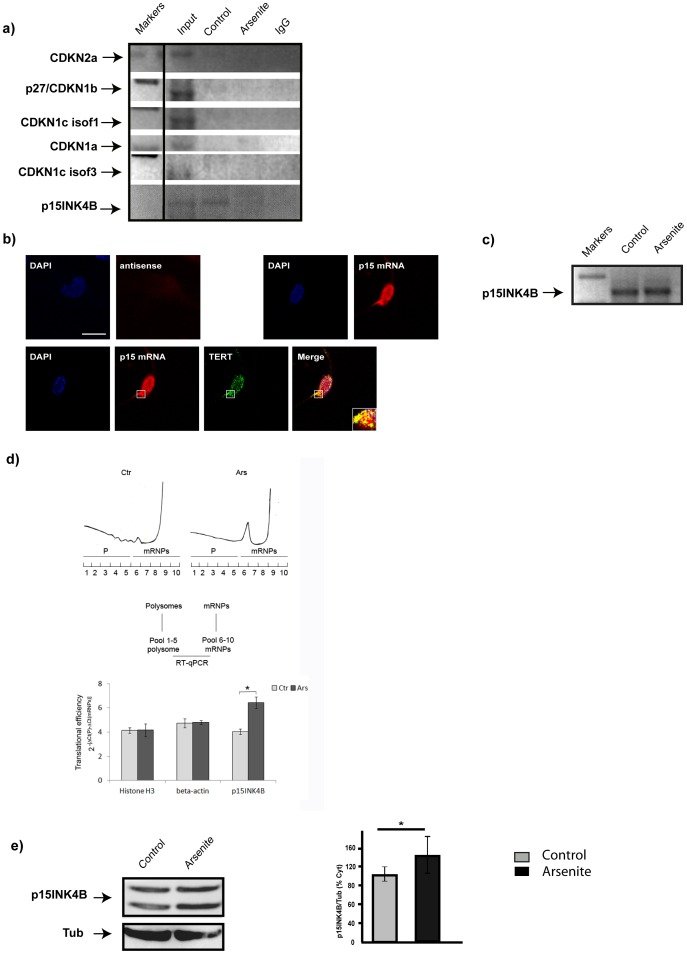
TERT granules contain the mRNA encoding the pro-survival cyclin inhibitor p15INK4B. **a)** TERT RNA-immunoprecipitation from cells under control or arsenite-induced stress: immunoprecipitated RNA was used for RT-PCR with specific primers for cell cycle regulators. Note that the only positive amplification product corresponds to the p15INK4B messenger, in the control but not stressed neurons (n = 2). **b) Upper panel.** p15INK4B in situ hybridization, negative control (anti-sense) and p15INK4B specific probe. Only the specific probe gives a signal, in the nucleus (DAPI positive) and in the cytoplasm. **Lower panel.** p15INK4B in situ hybridization (red) together with TERT immunofluorescence microscopy (green); nuclear labeling with DAPI (blue). Colocalization is evident in the perinuclear region (arrows in overlay image, “merge”) (n = 3). **c)** p15INK4B mRNA levels in 10 DIV hippocampal neurons in culture, under control or arsenite treatment. Arsenite does not result in degradation of the messenger (n = 3). **d)** Representative A254 gradient profile of control (Ctr) and arsenite stressed neurons (Ars); translational efficiency of p15INK4B mRNA was normalized to Histone 3 and β-actin (beta-actin) mRNA, as measured by RT-qPCR assay, using the following algorithm: 2-[ΔCt(P)- ΔCt(mRNPs)]. Stress induces p15INK4B translocation to the polysomes, reflecting higher translation. Standard errors are shown (n = 3). **e)** Western blot analysis of p15INK4B from 10 DIV hippocampal neurons in culture, in control and in neurons treated with arsenite. Tubulin is used as loading control. Note that arsenite increases the levels of p15INK4B. Bar graph on the right is the quantification of this experiment (means ± the s.d. of three different cultures; *p<0.05).

After the last division, neurons acquire biochemical, physiological and morphological properties that make them remain arrested in the G0 phase for the rest of their lives. Nonetheless, many cell cycle control proteins become up-regulated in the aging brain[26]. It is thought that the reactivation of the cell cycle is a consequence of stress accumulation and that it can lead to apoptotic signaling[27]. Thus, we tested whether TERT associates with mRNAs encoding cell cycle components and discovered that the TERT granules contain mRNA for p15INK4B (Fig. 3a). This observation is in agreement with the computational prediction of G-quadruplex structures in this RNA. The association of TERT with p15INK4B in RNA granules was confirmed in a double in situ hybridization-immunofluorescence staining experiment (as in Zalfa *et al.*,[28]) (Fig. 3b). Note however, that arsenite treatment resulted in the loss of the messenger from the TERT complex, suggesting that stress induces the release of the messenger, either for translation or for degradation. To distinguish between these two possibilities, we performed RT-PCR using total RNA purified from hippocampal neurons, both control and treated with arsenite. The p15INK4B mRNA was still amplified (Fig. 3c), thus excluding degradation and supporting higher translation. In fact, q-PCR from polysome gradients of control and arsenite treated neurons revealed a 40% increase in the translational efficiency of p15INK4B messenger (Fig. 3d). Western blot analysis of the same cell extracts showed the corresponding increase in p15INK4B protein levels (Fig. 3e).

**Figure 4:**
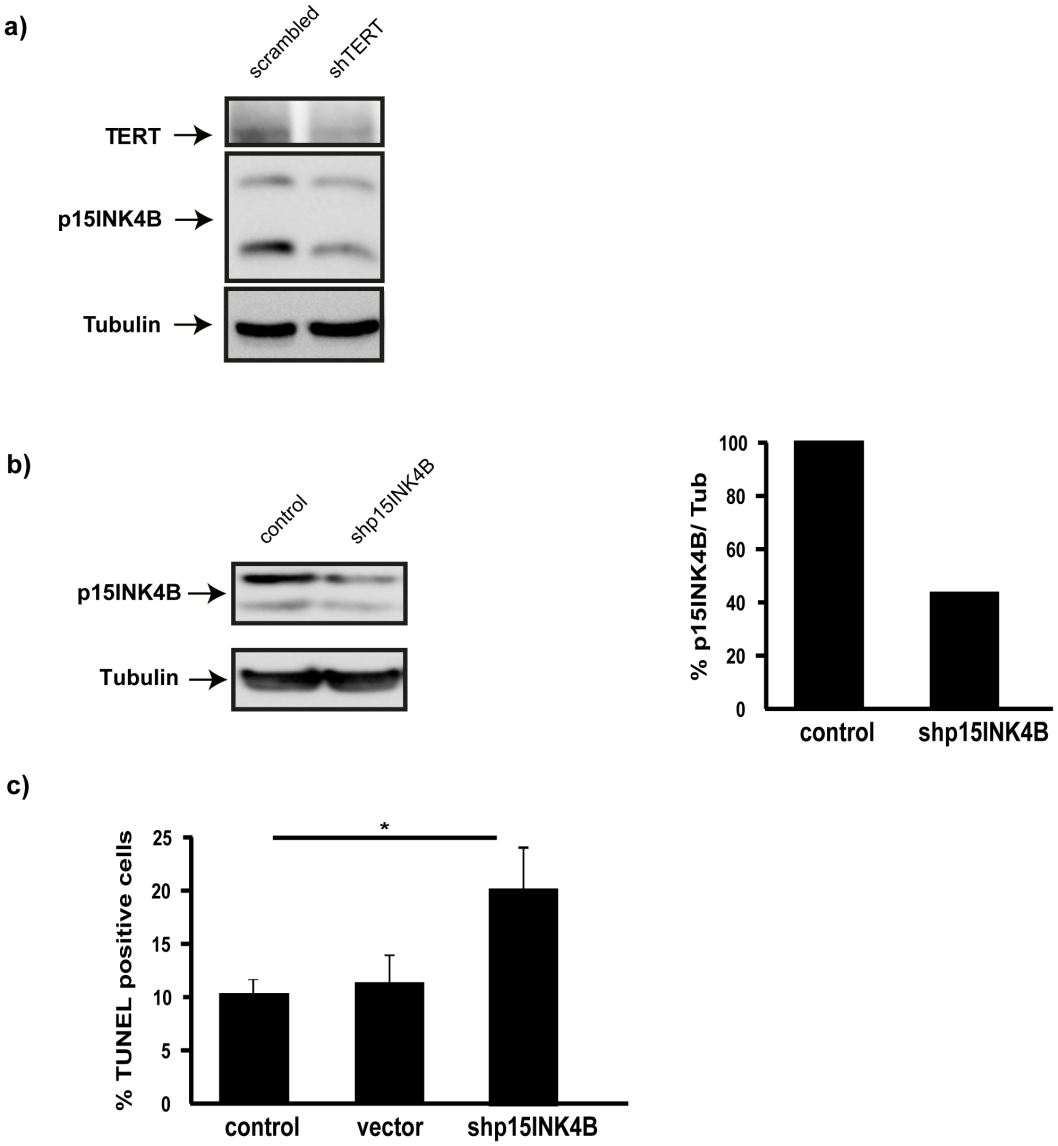
TERT exerts its anti-apoptotic role through regulation of p15INK4B messenger, a**)** Western blot analysis of TERT and p15INK4B levels in 10 DIV hippocampal neurons infected with scrambled or shTERT; Tubulin is used as loading control. Note that p15INK4B levels are reduced by TERT downregulation (n = 2). **b)** Western blot analysis of p15INK4B levels in control 10 DIV hippocampal neurons and infected with p15INK4B shRNA. The reduction in protein content is more than 50% (bar graph on the right, n = 2). **c)** Tunel assay of 10 DIV hippocampal neurons under control conditions (control) and after infection with an empty vector (vector) or with the shRNA for p15INK4B (shp15INK4B). The bar graph illustrates the significant cell death under this last condition (mean ± the s.d. of three different cultures; * p<0.05).

To test whether TERT could exert its pro-survival role (see S2 and S3) through the regulation of this inhibitor, we first knocked down TERT and analyzed the levels of p15INK4B. Consistent with this possibility, TERT down-regulation led to a significant decrease in this protein’s levels (Fig. 4a). Next, we directly addressed the pro-survival role by knocking down p15INK4B with a shRNA lentiviral vector (Fig. 4b). This experiment confirmed that low levels of p15INK4B increases the levels of apoptosis in cultured hippocampal neurons (Fig. 4c).

## Discussion

We have here demonstrated that neuronal aging is accompanied by the increased translocation of TERT from the nucleus to the cytoplasm. In hippocampal neurons in culture (see S1), TERT was exclusively nuclear in the early developmental stages (3 DIV) and abundant in the cytosol with time *in vitro*, especially 2 weeks after synaptogenesis, when metabolic demands are higher. We also observed cytoplasmic TERT in fully differentiated neurons *in situ*, indicating that TERT nucleus-to-cytoplasm change with age is a normal event in the biology of these cells. The increased levels of TERT in the cytosol of aged neurons may truly relate to a pro-survival need at this stage of life, as its knockdown resulted in higher apoptosis (see S1–S3). While it remains to demonstrate that this is also the case *in vivo*, our results strengthen the recent work by Eitan et al.[29] [30]. These authors found that the over-expression of TERT plays a protective role against oxidative stress in the brain and in motor neurons, delaying the onset and the progression of amyotrophic lateral sclerosis (ALS).

Second, our work shows that TERT is part of RNA granules in fully differentiated neurons. These RNA granules may well be a type of SGs. In fact, TERT co-precipitates and co-localizes with several components of SGs, including the ß-actin mRNA, P-elF2α, TIA1 and PABP. Moreover, the observation that TIA1 pulls down P-elF2α only in arsenite treated cells and TERT in both, stressed and non-stressed neurons, suggests the existence of two pools of TIA1-TERT complexes, with different composition. In support of this possibility, we could find only one of the two TIA1 mRNA targets in our TERT-IP experiment.

Mechanistically, TERT may be part of a type of RNA granules in which mRNAs are sequestered in order to prevent their degradation. This assumption comes from the observation that TERT downregulation results in the reduction of the amount of p15INK4B protein under basal levels of stress. Upon acute stress, TERT dissociates from this mRNA allowing its efficient translation, as demonstrated by qPCR in polysome gradient from stressed neurons. Under the stress condition, release from SGs does not lead to degradation of the p15INK4B mRNA possibly because of its transfer to the translation complex. Alternatively, degradation does not occur as part of the cells’ stress response (i.e. selective inhibition of RNA degradation). Regardless, our results may help to understand how the aging brain can resist the pressure of stressful stimuli that are imposed in an already physiologically stressed background. In this sense, our findings could explain the precocious aging observed in telomerase mutant mice[31]. However, precocious aging in TERT mutant mice may be due to more than the telomere elongation-independent mechanism. Recent work demonstrated that the aging phenotype in these mice occurs by the failure in proper telomere elongation[32]. Therefore, it is important that future work investigates whether a failure in the mechanism described herein is involved in the appearance of brain pathologies in elderly individuals.

## Supporting Information

Figure S1 **Cytoplasmic TERT increases with age in hippocampal neurons,** ***in vitro*** **and** ***in situ*** **. a)** Western blot analysis of TERT levels in the cytoplasmic and nuclear fraction of 3, 14 and 23 DIV hippocampal neurons. Primary cultures were prepared from Wistar rat fetuses at embryonic day 18–19 as described by Kaech and Banker[12]. Western Blotting: the soluble and the nuclear fractions from hippocampal neurons, were separated and loaded on poly-acrylamide gel. Note that TERT levels increase in the cytoplasm and decrease in the nucleus with time *in vitro*. Tubulin is used as loading control. Cdc2 and Histone 3 (Hist3) are used, respectively, as cytoplasmic and nuclear markers. Bar graph on the bottom represents the mean ± s.d. of three different cultures (*p<0.05). **b)** Western blot analysis of pJNK levels in the cytoplasm of 3, 14 and 23 DIV neurons. Note the parallelism between the increased cytoplasmic levels of TERT with the pJNK stress response. Total levels of JNK do not change with age in culture (n = 2). **c)** Representative confocal images of neurons stained for TERT (green) and counterstained with DAPI (blue) reveal the gradual accumulation of TERT in the cytoplasm with time *in vitro*. Bar: 10 µm. Bar graph on the bottom reflects the mean ± the s.d. of three different cultures. **d)** Representative images of brain slices from embryonic (E-17) adult (23 Months) mice stained with TERT (green), DAPI (blue) and Ctip2 (E-17) or NeuN (adult, red). Boxed area is enlarged in the right column of each panel: TERT is abundant in the cytosol of adult neurons (note exclusion from the nucleus of NeuN positive cells) and in the nucleus of embryonic neurons. Scale bar: 0.5 µm. (n = 3).
(TIF)

Figure S2 **TERT plays a pro-survival role in cultured hippocampal neurons. a)** Western blot analysis of total TERT from 10 DIV neurons, uninfected (control) or infected with scrambled shRNA or with a TERT shRNA (2) different from the one used for the experiments described in Fig. 2. This shRNA TERT lentivirus also led to significant reduction. **b)** Caspase 3 cleavage assay in neurons infected with 2 different shRNA TERT lentiviral particles**.** Note that reduced TERT levels are accompanied by increased caspase 3 cleavage product. Bar graph in the bottom of the panel highlights the difference (n = 3). **c)** Tunel assay of the experiment in **a** and **b**. Bars are the means ± the s.d. of three different cultures. *p<0.05.
(TIF)

Figure S3 **TERT plays a pro-survival role in a nucleus-to-cytoplasm transport process. a)** Western blot analysis of total TERT from control 10 DIV neurons or transfected with EGFP (vector) or the TERT cDNA together with the EGFP vector (cDNA). Tunel assay (right bar graph is the mean ± the s.d., corresponding to three different cultures, *p<0.05) reveals the anti-apoptotic effect of ectopic TERT. **b)** TERT expression in the cytosolic fraction of TERT overexpressing neurons (cDNA), TERT over-expressing neurons pre-treated with Leptomycin B (cDNA+LeptB) and EGFP vector with (vector+LeptB) or without (vector) pre-treatment with Leptomycin B. Leptomycin B reduces the pro-survival effect of TERT over-expressing neurons. Leptomycin B alone does not increase neuronal death significantly. Bar graph is the mean ± s.d. corresponding to three different cultures. *p<0.05. **c)** TERT levels in the cytosolic fraction of control neurons, stressed with hydrogen peroxide (H_2_O_2_) and stressed with Leptomycin B pre-treatment (H_2_O_2_+LeptB). Bar graph on the right shows the apoptosis **(**Tunel positive cells) assay of this experiment. Bar graph is the mean ± s.d. corresponding to three different cultures. *p<0.05. **d)** Cells were transfected with D-tomato and photoactivatable-EGFP-TERT. Positive transfection is shown in red; photoactivated TERT is in green. In control cells (Ctr) photoactivation reveals TERT in the nucleus. In stressed cells (H_2_O_2_), the photoactive protein is found in the cytoplasm. Bar: 5μm. Photoactivatable TERT GFP was made using the TERT cDNA (Imagenes) (n = 3).
(TIF)

Figure S4 **TERT antibody specificity assays.** The upper figure shows the concentration-dependent response of the TERT antibody in total extract from HEK cells. The lower panel shows the reduced signal of the antibody in neurons infected with two different shRNA (1 and 2) TERT lentiviral particles (left and middle panels). The right panel shows the increased in signal intensity to this antibody in neurons transfected with TERT cDNA.
(TIF)
