# Correction: Cytoplasmic TERT Associates to RNA Granules in Fully Mature Neurons: Role in the Translational Control of the Cell Cycle Inhibitor p15INK4B

**DOI:** 10.1371/annotation/e2d5d0e4-fb3a-4d53-a2de-99cd8d045d1c

**Published:** 2013-10-17

**Authors:** Francesca Iannilli, Francesca Zalfa, Annette Gartner, Claudia Bagni, Carlos G. Dotti

Two funding organizations and two grants were incorrectly omitted from the Funding Statement. The following should be included in the Funding Statement: "This work was also supported by Telethon GGP10150 and PRIN 2008 to CB." 

