# Supplementary figures and images for: Cytoplasmic TERT Associates to RNA Granules in Fully Mature Neurons: Role in the Translational Control of the Cell Cycle Inhibitor p15INK4B
Source: PLoS One. 2013 Jun 18;8(6):e66602. doi: 10.1371/journal.pone.0066602 (PMC3688952; doi:10.1371/journal.pone.0066602)

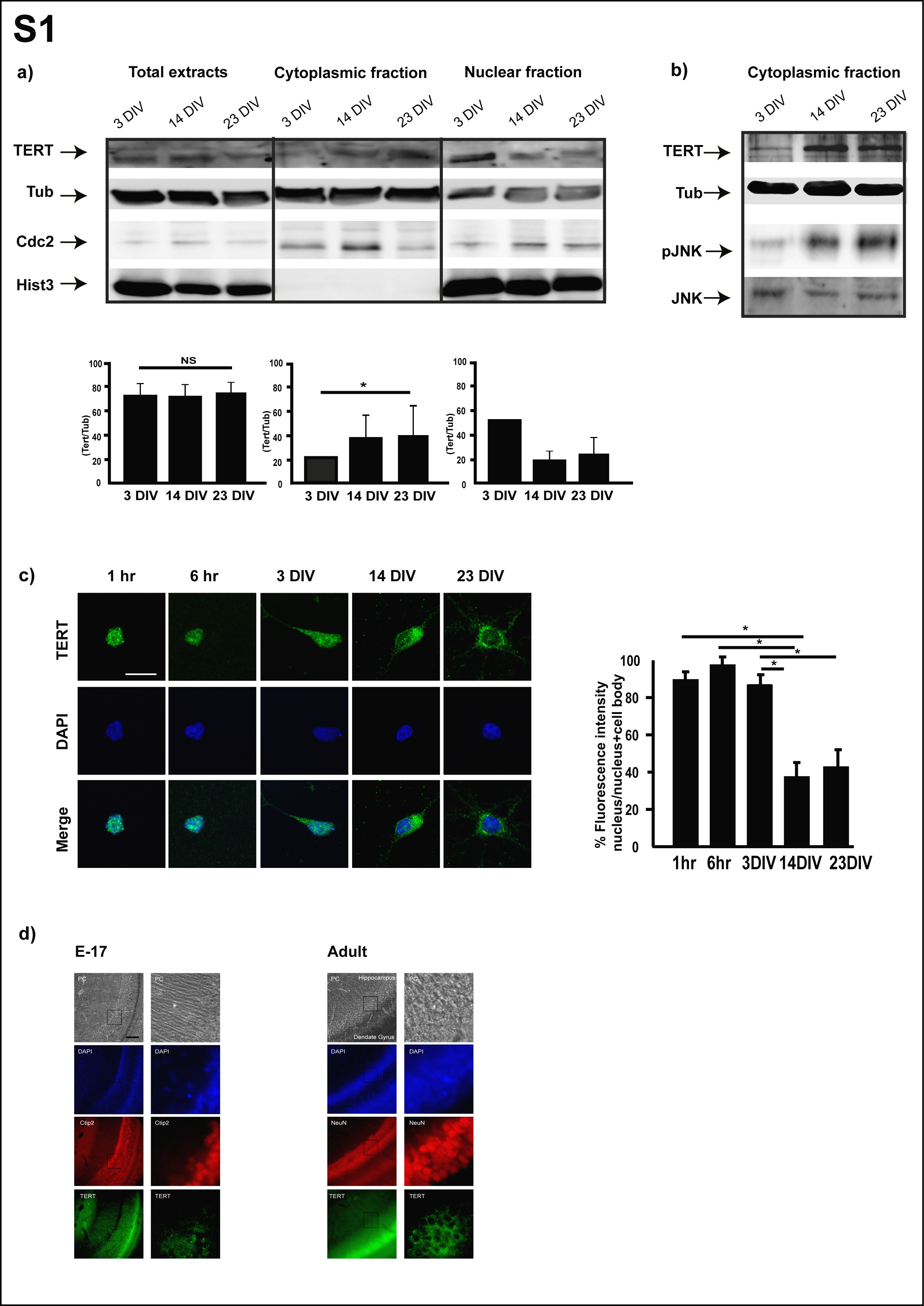

Supplement: Figure S1 — Cytoplasmic TERT increases with age in hippocampal neurons, in vitro and in situ . a) Western blot analysis of TERT levels in the cytoplasmic and nuclear fraction of 3, 14 and 23 DIV hippocampal neurons. Primary cultures were prepared from Wistar rat fetuses at embryonic day 18–19 as described by Kaech and Banker[12]. Western Blotting: the soluble and the nuclear fractions from hippocampal neurons, were separated and loaded on poly-acrylamide gel. Note that TERT levels increase in the cytoplasm and decrease in the nucleus with time in vitro. Tubulin is used as loading control. Cdc2 and Histone 3 (Hist3) are used, respectively, as cytoplasmic and nuclear markers. Bar graph on the bottom represents the mean ± s.d. of three different cultures (*p<0.05). b) Western blot analysis of pJNK levels in the cytoplasm of 3, 14 and 23 DIV neurons. Note the parallelism between the increased cytoplasmic levels of TERT with the pJNK stress response. Total levels of JNK do not change with age in culture (n = 2). c) Representative confocal images of neurons stained for TERT (green) and counterstained with DAPI (blue) reveal the gradual accumulation of TERT in the cytoplasm with time in vitro. Bar: 10 µm. Bar graph on the bottom reflects the mean ± the s.d. of three different cultures. d) Representative images of brain slices from embryonic (E-17) adult (23 Months) mice stained with TERT (green), DAPI (blue) and Ctip2 (E-17) or NeuN (adult, red). Boxed area is enlarged in the right column of each panel: TERT is abundant in the cytosol of adult neurons (note exclusion from the nucleus of NeuN positive cells) and in the nucleus of embryonic neurons. Scale bar: 0.5 µm. (n = 3). (TIF) [file pone.0066602.s001.tif]

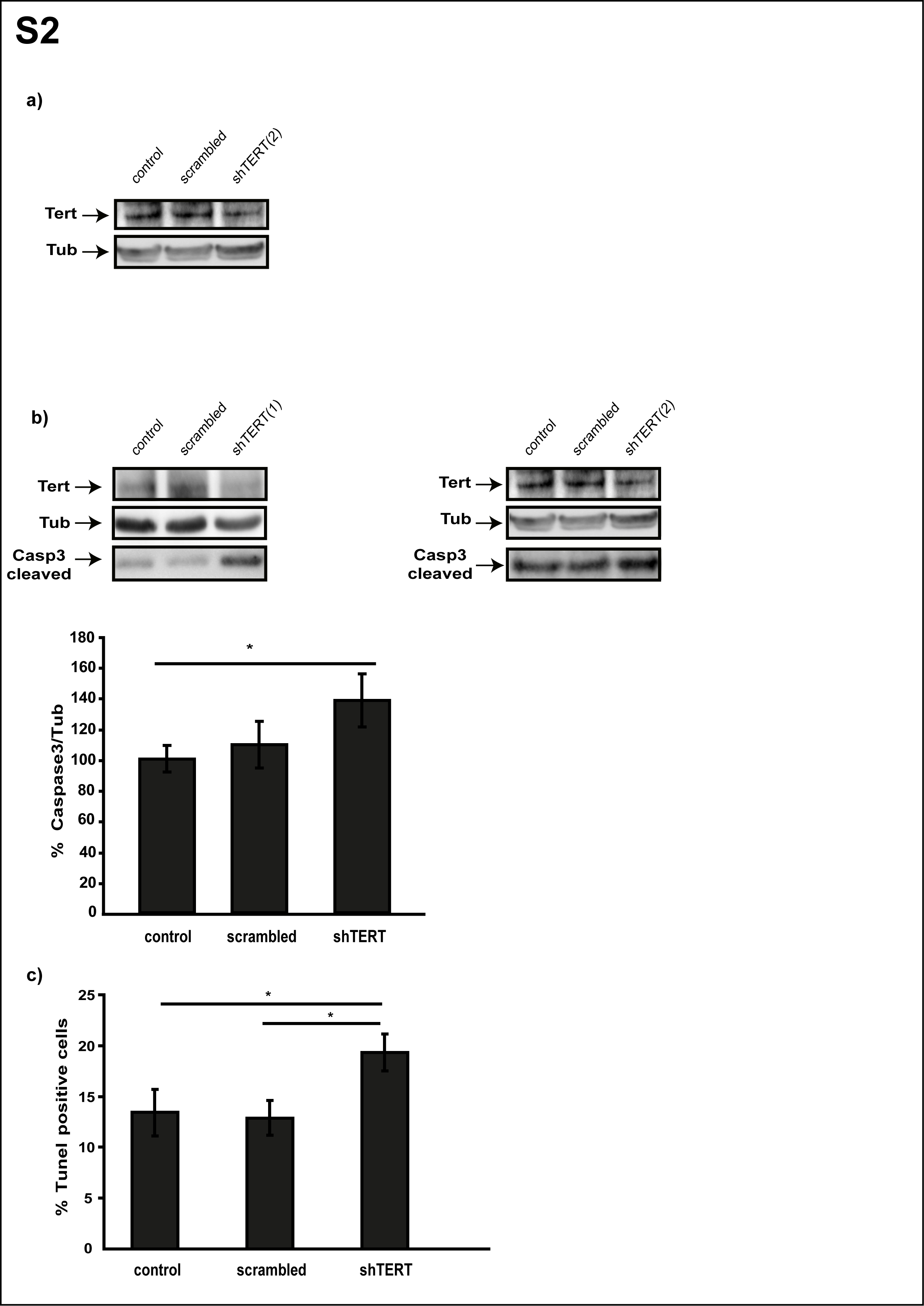

Supplement: Figure S2 — TERT plays a pro-survival role in cultured hippocampal neurons. a) Western blot analysis of total TERT from 10 DIV neurons, uninfected (control) or infected with scrambled shRNA or with a TERT shRNA (2) different from the one used for the experiments described in Fig. 2. This shRNA TERT lentivirus also led to significant reduction. b) Caspase 3 cleavage assay in neurons infected with 2 different shRNA TERT lentiviral particles. Note that reduced TERT levels are accompanied by increased caspase 3 cleavage product. Bar graph in the bottom of the panel highlights the difference (n = 3). c) Tunel assay of the experiment in a and b. Bars are the means ± the s.d. of three different cultures. *p<0.05. (TIF) [file pone.0066602.s002.tif]

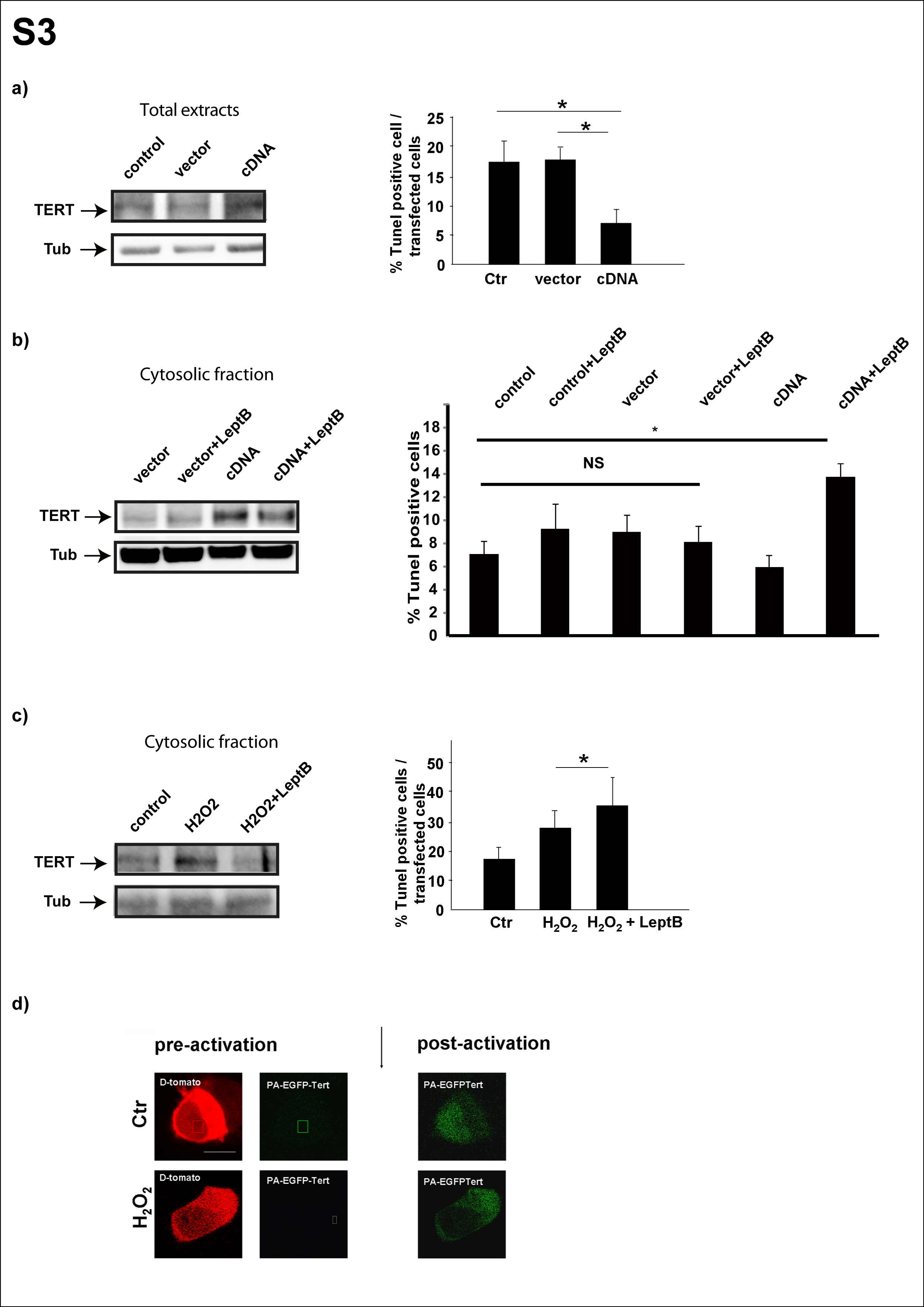

Supplement: Figure S3 — TERT plays a pro-survival role in a nucleus-to-cytoplasm transport process. a) Western blot analysis of total TERT from control 10 DIV neurons or transfected with EGFP (vector) or the TERT cDNA together with the EGFP vector (cDNA). Tunel assay (right bar graph is the mean ± the s.d., corresponding to three different cultures, *p<0.05) reveals the anti-apoptotic effect of ectopic TERT. b) TERT expression in the cytosolic fraction of TERT overexpressing neurons (cDNA), TERT over-expressing neurons pre-treated with Leptomycin B (cDNA+LeptB) and EGFP vector with (vector+LeptB) or without (vector) pre-treatment with Leptomycin B. Leptomycin B reduces the pro-survival effect of TERT over-expressing neurons. Leptomycin B alone does not increase neuronal death significantly. Bar graph is the mean ± s.d. corresponding to three different cultures. *p<0.05. c) TERT levels in the cytosolic fraction of control neurons, stressed with hydrogen peroxide (H2O2) and stressed with Leptomycin B pre-treatment (H2O2+LeptB). Bar graph on the right shows the apoptosis (Tunel positive cells) assay of this experiment. Bar graph is the mean ± s.d. corresponding to three different cultures. *p<0.05. d) Cells were transfected with D-tomato and photoactivatable-EGFP-TERT. Positive transfection is shown in red; photoactivated TERT is in green. In control cells (Ctr) photoactivation reveals TERT in the nucleus. In stressed cells (H2O2), the photoactive protein is found in the cytoplasm. Bar: 5μm. Photoactivatable TERT GFP was made using the TERT cDNA (Imagenes) (n = 3). (TIF) [file pone.0066602.s003.tif]

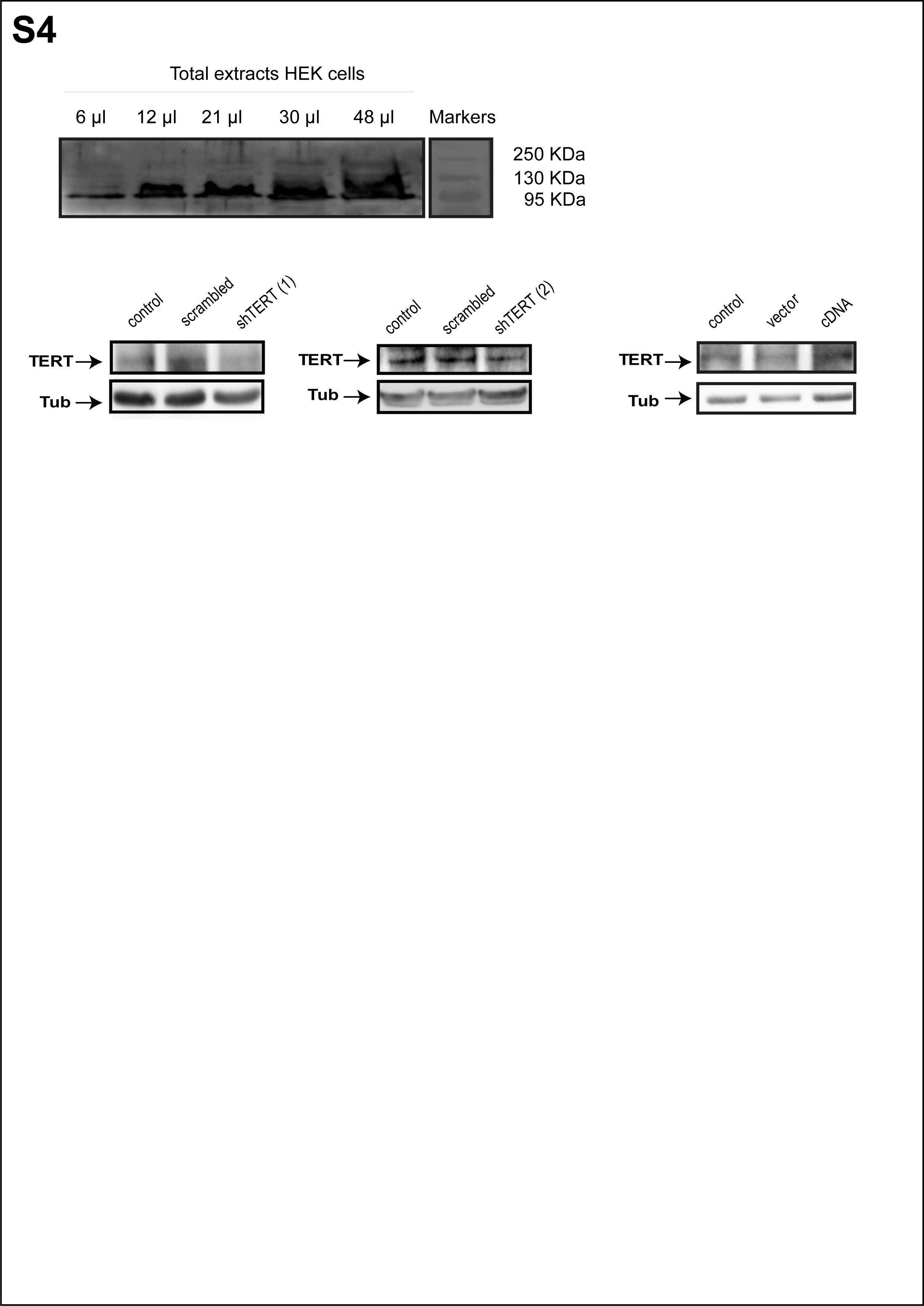

Supplement: Figure S4 — TERT antibody specificity assays. The upper figure shows the concentration-dependent response of the TERT antibody in total extract from HEK cells. The lower panel shows the reduced signal of the antibody in neurons infected with two different shRNA (1 and 2) TERT lentiviral particles (left and middle panels). The right panel shows the increased in signal intensity to this antibody in neurons transfected with TERT cDNA. (TIF) [file pone.0066602.s004.tif]
